# Sex differences in type 2 diabetes: an opportunity for personalized medicine

**DOI:** 10.1186/s13293-023-00571-2

**Published:** 2023-12-13

**Authors:** Meredith L. Johnson, Joshua D. Preston, Cetewayo S. Rashid, Kevin J. Pearson, J. Nina Ham

**Affiliations:** 1 Department of Surgery, University of Kentucky College of Medicine, Lexington, KY 40506 USA; 2 Medical Scientist Training Program, Emory University School of Medicine, Atlanta, GA 30322 USA; 3 Nutrition and Health Sciences, Laney Graduate School, Emory University, Atlanta, GA 30322 USA; 4 Department of Pharmacology and Nutritional Sciences, University of Kentucky College of Medicine, Lexington, KY 40536 USA; 5 Division of Pediatric Endocrinology, Diabetes, and Metabolism, Columbia University Vagelos College of Physicians and Surgeons, New York, NY 10032 USA

**Keywords:** Sex difference, Type 2 diabetes, Sexual dimorphism, Obesity, Chronic complications, Detection, Diagnosis, Therapy

## Abstract

Over the past several decades, substantial ground has been gained in understanding the biology of sex differences. With new mandates to include sex as a biological variable in NIH-funded research, greater knowledge is forthcoming on how sex chromosomes, sex hormones, and social and societal differences between sexes can affect the pathophysiology of health and disease. A detailed picture of how biological sex impacts disease pathophysiology will directly inform clinicians in their treatment approaches and challenge canonical therapeutic strategies. Thus, a profound opportunity to explore sex as a variable in personalized medicine now presents itself. While many sex differences are apparent in humans and have been described at length, we are only beginning to see how such differences impact disease progression, treatment efficacy, and outcomes in obesity, type 2 diabetes, and cardiovascular disease. Here, we briefly present the most salient and convincing evidence of sex differences in type 2 diabetes detection, diagnostics, disease course, and therapeutics. We then offer commentary on how this evidence can inform clinicians on how to approach the clinical workup and management of different patients with diabetes. Finally, we discuss some gaps that remain in the literature and propose several research questions to guide basic and translational researchers as they continue in this growing area of scientific exploration.

## Highlights

- Evidence continues to emerge regarding sex differences in type 2 diabetes, yet most clinical practice has not adapted to consider such differences.
- We sought to briefly describe the strongest evidence on sex differences in type 2 diabetes care and discuss how this may impact research and clinical practice.
- Data from the published literature demonstrate sex differences in the detection, diagnostics, and disease course of type 2 diabetes, as well as some minor differences in therapy.
- While further investigation and larger trials are needed, quality of care and outcomes in type 2 diabetes will be improved if sex-specific treatment strategies are developed.

## Plain language summary

For decades, most research in the laboratory and clinical settings focused primarily on males. However, more recently, grant-funding agencies, including the National Institutes of Health, have prioritized research that studies both males and females. This has dramatically improved our understanding of how biological sex impacts whether a person is at higher risk for developing a particular disease and what treatment options may be best to achieve the healthiest outcomes. This article offers the perspectives of practicing physicians and scientists on how our knowledge about biological sex may impact disease incidence, progression, treatment options, and outcomes in obesity, diabetes, and heart disease. The piece will offer a broad overview of the current science and personalized medicine approaches in these areas. It then discusses gaps in our knowledge and proposes several questions to guide future research.

## Introduction

The last decade has been a period of further investigation of biological sex differences in human disease course, treatment, and prevention. This change was partially ushered in by a 2014 NIH mandate  that required the inclusion of sex as a biological variable in the analysis of all future NIH-funded research. As a result, sex differences in cardiometabolic diseases have become a burgeoning area of research. Sex differences in whole-body metabolism, energy balance, and body composition are a well-observed phenomenon across the animal kingdom; however, it has only been in the last several decades that the mechanistic underpinnings (i.e., chromosomal, hormonal, etc.) have been better explained.

From an epidemiological perspective, obesity, diabetes, and cardiovascular disease fundamentally affect males and females at different times and in varying ways. In a recent review, Tramunt et al. [1] outline these differences at length, not the least of which are the predisposition for men to develop type 2 diabetes (T2D), the protective role of sex hormones in metabolic health, and the distinct differences in glucose and lipid handling between males and females. These seemingly obvious differences beg the question: could the sex of a patient be a factor used to guide prevention, detection, and/or therapy for T2D? At this time, there are not sufficient data to firmly support evidence-based clinical recommendations for the management of T2D based on biological sex; however, we do foresee a future wherein a patient’s sex could assist in providing tailored, precise T2D screening, prevention, and care. There are examples from various fields that have successfully generated sex-based recommendations, cardiovascular medicine being one of the most notable (see Tannenbaum, et al. [2] for a framework on using sex to develop recommendations and guide clinical practice).

We provide a brief comment on potential sex-based targets within T2D detection, diagnostics, disease course, and therapy. Our literature search was based on keywords, and we selected pertinent and recent literature on these topics directed toward both scientific and clinical audiences. This commentary was not meant to be a systematic review, and articles were selected that provided a well-rounded view of the current state of the literature, research, and treatment in sex differences in diabetes care. We aim at addressing only that which is pathophysiologic in nature and do not comment on gender differences, which are more nuanced social constructs. Due to the breadth of the existing literature, we have referred to expanded reading on topics where a comprehensive summary could not be provided. Overall, the primary purpose of this article is to serve as a call to action and to provide researchers, clinicians, and medical societies with the impetus for the development and execution of sex-based strategies and guidelines in T2D care and management.

## Main text

### Detection, diagnostics, and disease course

In our review of the literature, we found evidence for appreciable differences in the disease course and pathophysiology of T2D between men and women and, as such, see opportunities for more precise detection, diagnostics, and interventions by including a sex-based risk stratification. First, at diagnosis of T2D, women were on average 3 years older than men and had higher mean BMI, cholesterol, and lower hemoglobin A1c (HbA1c), contributing to the idea that the timing of screening is an important step in the development of sex-based care [3]. Moreover, approximately two-thirds of children and adolescents diagnosed with type 2 diabetes are female, underscoring the fact that females have higher rates of type 2 diabetes in youth compared to males who have a higher prevalence in midlife [4].

Evidence demonstrating the clear superiority of any one diagnostic test for males vs. females is lacking, though differences in diagnostic tests between the sexes have been demonstrated. For example, a number of biomarkers can more robustly predict T2D in women compared to men [5]. Moreover, the use of HbA1c alone as a diagnostic tool is problematic, given that it likely underpredicts fasting plasma glucose (FPG) in men [6]. The use of a single diagnostic approach in T2D ignores individual heterogeneity in pathogenesis. Furthermore, it also fails to account for potential sex-specific T2D etiologies, namely, peripheral insulin resistance (characterized by impaired glucose tolerance) in women vs. hepatic insulin resistance (characterized by elevated FPG) in men. Indeed, some have argued for the superiority of OGTT in detecting T2D in women and FPG in men [7]; though, implementing this approach may necessitate personalized doses based on BMI, lean mass, or body surface area, given the influence of anthropomorphic parameters on OGTT results when a universal 75 g glucose load is administered [8]. While more research is warranted to determine exactly which models could best predict disease presence in each sex, this example indicates that by selecting tailored diagnostic parameters based on the patient’s sex, patients could be more accurately diagnosed. Regardless, it is clear that unidimensional approaches for diagnosis are inadequate, and we advocate for the adoption of a systematic and personalized method of diagnosis described by Abdul-Ghani and DeFronzo [9], which, importantly, would have the ability to accurately diagnose T2D and inform clinicians of its etiology, irrespective of sex. It is critical to improve diagnostic methods to recognize T2D as early as possible given the “legacy effect” of hyperglycemia and the clear benefits of timely diagnosis and early, aggressive treatment in forestalling complications in both males and females [9, 10].

Tailored monitoring for the complications to which males and females are differentially susceptible can also function as a precision medicine tool for the prevention of diabetes complications. Vascular complications are a leading cause of morbidity and mortality in T2D. In general, men tend to suffer more from peripheral arterial and microvascular complications of diabetes, whereas women tend toward thromboembolic and macrovascular complications [11, 12]. Women with type 2 diabetes have a 27% higher stroke risk and a 19% higher vascular dementia risk than men [13]. The onset of complications is generally later in women (especially postmenopausal), yet the severity and pathological progression of these complications are more intense than in men, who seem to have a more linear progression. In a healthy population, coronary artery disease (CAD) generally develops around a decade later in women vs. men, suggesting that ovarian hormones in this setting are cardioprotective [14]. However, diabetes appears to abolish female protection from CAD, with most evidence supporting that diabetes confers a greater risk for CAD death in women compared with men [4]. This implies an insidious onset of T2D complications in females, arguing for careful and early surveillance in females with aggressive treatment to prevent heart damage before it is too late.

### Therapeutics

The ABCDE (Age, Body weight, Complications and Comorbidities, Duration of diabetes, and life Expectancy) approach described by Abdul-Ghani and DeFronzo provides a rigorous framework for personalized diabetes treatment [9]. In our review of the literature, we found the evidence for sex-based indications for T2D pharmacological therapy to be incomplete. However, the strongest existing evidence shows a generally improved response in women to thiazolidinediones (TZDs), which function as insulin sensitizers, and greater responses in men to sulfonylureas, which augment insulin secretion. Dennis et al. examined data from both cohort and randomized control trials (RCTs) and found improved HbA1c response to sulfonylureas over TZDs in males with obesity, while TZDs more effectively reduced HbA1c in females with obesity [15]. This study provides evidence to encourage updating clinical practice toward tailoring multidrug diabetes treatment, though we do recognize these are not first-line drug choices currently. It is important to note the strategy employed by this study, namely, the subgroup stratification by sex and BMI. While a simple analysis of these data may yield no difference in drug response by sex, the subgroup analysis reveals stark differences in drug response. Such a paradigm should be taken into consideration with future studies and practice guidelines, especially when evaluating subgroup effects of newer diabetes drugs that are increasing in use, such as SGLT2 inhibitors and GLP-1 receptor agonists. Though the data are still nascent, female sex appears to be an independent factor linked to greater weight loss achievement after treatment with GLP-1 receptor agonists [16]. This is also the case for adverse events (primarily gastrointestinal) resulting from the use of these medications, which appear in higher percentages in women. Regarding SGLT2 inhibitors, limited data may indicate improved efficacy in lowering HbA1c in males [17]. It is important to note that the recent FDA approval of empagliflozin for the treatment of heart failure with preserved ejection fraction (HFpEF) will provide a high volume of data to analyze any sexual dimorphism in response to this drug class, whether for the primary indication of HFpEF or for secondary effects on glycemic control, given the frequent co-incidence of heart failure and T2D. Mixed results have been observed in the effectiveness of insulin therapy in male vs. female patients with T2D, with various studies displaying improved responses in males or females but some showing no differences [18–24]. Taken together, we argue, along with Regensteiner and Reusch [25], for the critical role of RCTs of diabetic therapies, which examine sex as a primary, independent variable, as current data are largely confined to retrospective analysis. For an expanded discussion on sex differences in cardiovascular consequences of metabolic disease, we recommend Regensteiner and Reusch’s [25] recent review, and for a comprehensive presentation of sex differences in T2D pharmacotherapy, see Kautzky-Willer et al. [7].

While the above evidence should be applied loosely until further study is completed, we do believe one of the sex-based T2D treatment opportunities with the most robust evidence lies in hormone replacement therapy (HRT). In hypogonadal men with prediabetes, testosterone (T) supplementation has been shown, among several cardiometabolic parameters, to be remarkably effective in preventing progression to diabetes. Indeed, T supplementation in such patients provides a staggering improvement in all-cause mortality [26]. In addition, TZDs decrease bioavailable T in men and cause weight gain [27]. Given the common association of obesity and hypogonadism in men, as well as the increased aromatization of testosterone to estrogen in obesity, this sex-based demographic serves as an obvious example of a group in which treatment can be targeted to improve outcomes. Considering sex differences related to sulfonylurea response and the fact that T itself augments insulin secretion [28], T supplementation may be indicated for men with T2D, especially those with concurrent obesity, although the hypogonadal status of the patient should be given great consideration before initiating HRT.

For women, the debate over menopausal HRT therapy (MHRT) with estrogen has persisted for decades. There are a variety of metabolic benefits of MHRT, and MHRT can decrease the risk of T2D development or progression in perimenopausal women [29]. Part of the complexity of MHRT in the context of metabolic disease is the interconnected risks and benefits of MHRT and how those may change with hormone replacement. For example, T2D alone could be a contraindication for MHRT if the patient has concomitant cardiovascular risk factors, and yet, MHRT could potentially improve T2D, thus lowering cardiovascular risk and thereby “reopening” the appropriateness of MHRT for the same patient. Complicating the picture further, however, are the various routes of administration (i.e., oral vs. transdermal), along with the risks and benefits each carries in the setting of obesity and diabetes. Oral estrogens provide stronger anti-diabetogenic effects vs. transdermal but also confer a higher risk for thromboembolic events [29]. Since obesity and diabetes are commonly comorbid conditions, close attention must be given to selecting the route of administration that considers a patient’s risk factors for cardiovascular disease. Instead of seeing risk factors as a barrier to care, we believe this, and other previously discussed patient factors, are an opportunity for precision medicine to provide a treatment plan tailored to each patient. We commend the North American Menopause Society guidelines for MHRT for a formal algorithm to determine if MHRT is appropriate [30] and Mauvais-Jarvis et al. [29] for a thorough discussion of the topic. Such complex, interwoven factors must be taken into careful consideration when treating the perimenopausal female patient with diabetes or prediabetes.

### Perspectives and significance

Given the brief nature of this article, we are not able to discuss at length some emerging areas of research on sex differences in adipose tissue and skeletal muscle biology, the effect of sex hormones on whole-body energy metabolism and partitioning, emerging discoveries on the role of estrogen receptors in glucose uptake and metabolism, and the growing body of knowledge on chromosomal vs. hormonal influences on obesity, diabetes, and atherosclerosis. Moreover, we were unable to fully cover the unique considerations for diabetes risk and management for patients with PCOS or gestational diabetes mellitus, as well as for transgender patients undergoing gender-affirming hormone therapy. We urge clinicians and scientists alike to target research efforts and inquiries toward the above-mentioned areas going forward.

**Fig. 1:**
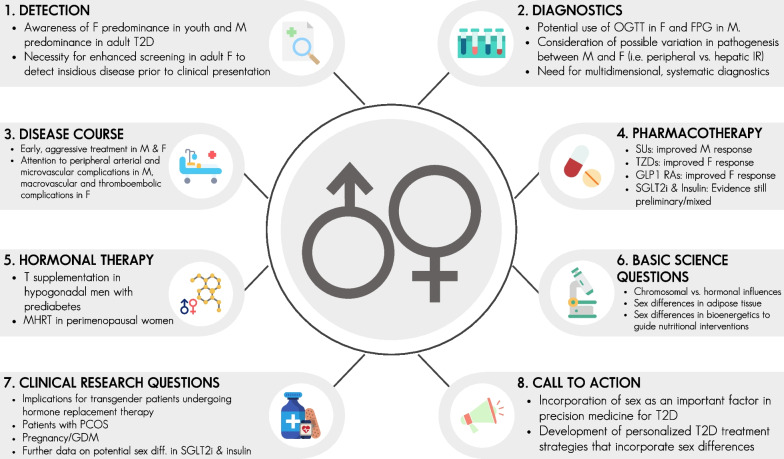
Graphical summary of sex differences in diabetes care and research. Abbreviations: *AT* adipose tissue, *F* female, *FPG* fasting plasma glucose, *GDM* gestational diabetes mellitus, *GLP-1 RA* glucagon-like peptide-1 receptor agonist, *IR* insulin resistance, *MHRT* menopausal hormone replacement therapy, *OGTT* oral glucose tolerance test, *PCOS* polycystic ovarian syndrome, *M* male, *SGLT2i* sodium–glucose cotransporter-2 inhibitor, *SU* sulfonylurea, *T* testosterone, *T2D* type 2 diabetes, *TZD* thiazolidinedione

We believe there is reason to be optimistic about the future use of patient sex in optimizing the precision of obesity and T2D care. The topics briefly mentioned in this paper should serve as starting points for clinical and basic science investigators to further execute definitive studies to inform clinical practice (Fig. 1). While further study is required to establish evidence-based practices, a number of salient preliminary clinical recommendations have been proposed in the literature [7]. Moreover, it is our perspective that the following areas warrant further investigation to gain a clearer understanding of the biology underlying sex differences in T2D:

- Determining hormonal vs. chromosomal contributions to the sex differences in cardiometabolic diseases. Novel animal models to this end have been described with intriguing results [31].
- Establishing whether sex differences in adipose tissue structure, function, and depots [32] have therapeutic implications. For example, determining if therapeutic strategies to induce beiging of adipose tissue [33] or reduce adipose tissue inflammation will result in improved glucose homeostasis in women vs. men.
- Examining if sex differences in bioenergetics are grounds for guided nutritional interventions in patients with T2D. It is reasonable to suggest that sex differences in lipid and glucose metabolism could lead to differential efficacy in dietary regimens, though much further study is needed to apply this concept clinically.

In the quest for precision medicine, it is easy to dive directly into complicated patient measures when determining treatment. Consistent with this, there is little to show in T2D care for personalized medicine efforts involving pharmacogenetic or other -omics-based biomarkers [9]. With the well-intentioned use of powerful technology to personalize treatment, we may neglect as “simplistic” the more basic patient variables, which are just as important for personalized medicine. Indeed, accounting for patient sex, age, race, clinical history, or phenotypic measures may be just as impactful as -omics or complex imaging. With a disease as burdensome and prevalent as T2D, we must consider every possible factor and variable in developing and enacting treatment strategies. We hope to see biological sex emerge as one of those factors in the coming decades.

## Data Availability

Not applicable.

## Abbreviations

ABCDE: Age, body weight, complications and comorbidities, duration, expectancy of life
BMI: Body mass index
CAD: Coronary artery disease
FPG: Fasting plasma glucose
GLP-1: Glucagon-like peptide-1
HbA1c: Hemoglobin A1c
HFpEF: Heart failure with preserved ejection fraction
HRT: Hormone replacement therapy
MHRT: Menopausal hormone replacement therapy
NIH: National Institutes of Health
OGTT: Oral glucose tolerance test
PCOS: Polycystic ovarian syndrome
RCTs: Randomized control trials
SGLT2: Sodium–glucose cotransporter-2
T: Testosterone
T2D: Type 2 diabetes
TZD: Thiazolidinedione
